# Veterinary Journalism

**Published:** 1896-06

**Authors:** 


### EDITORIAL.

### VETERINARY JOURNALISM.

The *Veterinary Magazine* for April comes to us in a new dress, and the improved appearance is very noticeable. The adoption of a highly finished paper and the change of type add greatly to its attractiveness, and now that it is fully up on time with its numbers we bespeak for it a strong place among the readers of veterinary periodicals in our country.

The *American Veterinary Review* for May comes to its readers in a more attractive form than ever, and exhibits a determination to fully sustain its long and prosperous career. A slight change in the color of the cover-pages, a more highly finished paper and new type of a very readable size will add greatly to the pleasure of its many readers. The great development in the journalism of our profession during the past two years bids fair to extend our usefulness and power to wider fields, and it is hoped that these journalistic efforts will receive suitable recognition. The JOURNAL takes to itself a part of the credit for this general improvement all along the line, and though it has all taken place at a time when the support of journals is least promising, it shows a determination to win support by deserving it.

